# Supplementary material for: Characterizing Seasonal Radial Growth Dynamics of Balsam Fir in a Cold Environment Using Continuous Dendrometric Data: A Case Study in a 12-Year Soil Warming Experiment
Source: Sensors (Basel). 2022 Jul 9;22(14):5155. doi: 10.3390/s22145155 (PMC9315762; doi:10.3390/s22145155)
Supplement: Supplementary file 1 [file sensors-22-05155-s001.zip › sensors-1772292-supplementary.pdf]

Supplementary material

Table S1. Growth onset (DOY; 5% of yearly growth completed) for control and soil-heated balsam fir trees over a 12-yr period (2010–2021).

**Trees**							**Years**							**Means**
		**2010**	**2011**	**2012**	**2013**	**2014**	**2015**	**2016**	**2017**	**2018**	**2019**	**2020**	**2021**	
**Control**	**101**	145	154	150	144	156	148	154	160	153	165	163	145	153
	**105**	146	157	147	148	138	151	157	163	165	168	163	146	154
	**111**	156		155										156
	**114**	145	154	143	133	164	147	150	150	152	162	150	146	150
	**121**	145	154	143	133	137	150	158	151		162	151	146	148
	**125**	145	154	143										147
**Heated**	**102**	145	169	151										155
	**104**	145	158	146	153	163	150	155	155	156	162	163	145	154
	**112**	145	154	146	143	154	148	152	153	152	162	152	145	151
	**115**	145	158	146										150
	**122**	145	159	150	143	154	147	151	150		162	151		151
	**124**	145	159											152
**Means**														
**Control**		147	155	147	140	149	149	155	156	157	164	157	146	151
**Heated**		145	160	148	146	157	148	153	153	154	162	155	145	152
**Overall**		146	157	147	142	152	149	154	155	156	163	156	146	152

Table S2. Growth cessation (DOY; 95% of yearly growth completed) for control and soil-heated balsam fir trees over a 12-yr period (2010–2021).

**Trees**		**Years**												**Means**
		**2010**	**2011**	**2012**	**2013**	**2014**	**2015**	**2016**	**2017**	**2018**	**2019**	**2020**	**2021**	
**Control**	**101**	268	229	224	227	289	226	264	235	283	276	238	206	247
	**105**	258	233	229	296	292	227	297	289	285	268	277	217	264
	**111**	198		286										242
	**114**	251	234	223	216	245	224	239	231	313	248	220	222	239
	**121**	271	233	253	226	265	288	255	235		245	233	211	247
	**125**	249	231	199										226
**Heated**	**102**	273	248	225										249
	**104**	198	209	230	257	292	213	255	218	234	247	246	206	234
	**112**	252	233	224	222	246	224	297	217	234	231	216	206	234
	**115**	272	234	309										272
	**122**	257	233	224	247	246	240	253	235		247	233		242
	**124**	273	234											254
**Means**														
**Control**		249	232	236	241	273	241	264	248	294	259	242	214	248
**Heated**		254	232	242	242	261	226	268	223	234	242	232	206	240
**Overall**		252	232	239	242	268	235	266	237	270	252	238	211	244

Table S3. Growing season duration (days; from 5% to 95% of yearly growth completed) for control and soil-heated balsam fir trees over a 12-yr period (2010–2021).

**Trees**		**Years**												**Means**
		**2010**	**2011**	**2012**	**2013**	**2014**	**2015**	**2016**	**2017**	**2018**	**2019**	**2020**	**2021**	
**Control**	**101**	123	75	74	83	133	78	110	75	130	111	75	61	94
	**105**	112	76	82	148	154	76	140	126	120	100	114	71	110
	**111**	42		131										87
	**114**	106	80	80	83	81	77	89	81	161	86	70	76	89
	**121**	126	79	110	93	128	138	97	84		83	82	65	99
	**125**	104	77	56										79
**Heated**	**102**	128	79	74										94
	**104**	53	51	84	104	129	63	100	63	78	85	83	61	80
	**112**	107	79	78	79	92	76	145	64	82	69	64	61	83
	**115**	127	76	163										122
	**122**	112	74	74	104	92	93	102	85		85	82		90
	**124**	128	75											102
**Means**														
**Control**		102	77	89	102	124	92	109	92	137	95	85	68	96
**Heated**		109	72	95	96	104	77	116	71	80	80	76	61	88
**Overall**		106	75	92	99	116	86	112	83	114	88	81	66	93

Table S4. Total annual growth (µm) for control and soil-heated balsam fir trees over a 12-yr period (2010–2021).

**Trees**		**Years**												**Means**
		**2010**	**2011**	**2012**	**2013**	**2014**	**2015**	**2016**	**2017**	**2018**	**2019**	**2020**	**2021**	
**Control**	**101**	1715	2015	1658	1117	1042	1133	1312	1202	1187	1289	982	1376	1336
	**105**	1327	1285	957	427	435	535	700	849	670	704	634	571	758
	**111**	542		430										486
	**114**	2148	1773	1675	566	194	448	766	1491	1463	1143	1421	1354	1203
	**121**	1449	1241	1109	606	518	623	806	1628		1085	1370	959	1036
	**125**	2251	2047	1733										2010
**Heated**	**102**	532	295	411										412
	**104**	1505	1251	1597	737	793	961	1251	1505	1267	1226	1159	1167	1202
	**112**	1258	1476	1240	946	937	949	1061	1434	1551	1116	1394	1013	1198
	**115**	652	964	826										814
	**122**	1620	1592	1323	789	723	1994	2228	2932		2170	2088		1746
	**124**	543	605											574
**Means**														
**Control**		1572	1672	1260	679	547	685	896	1292	1107	1055	1102	1065	1115
**Heated**		1018	1031	1079	824	818	1301	1513	1957	1409	1504	1547	1090	1216
**Overall**		1295	1322	1178	741	663	949	1160	1577	1228	1248	1293	1073	1160

Table S5. Average daily growth rate (µm/day) for control and soil-heated balsam fir trees over a 12-yr period (2010–2021).

Trees		Years												Means
		**2010**	**2011**	**2012**	**2013**	**2014**	**2015**	**2016**	**2017**	**2018**	**2019**	**2020**	**2021**	
**Control**	**101**	13.9	26.9	22.4	13.5	7.8	14.5	11.9	16.0	9.1	11.6	13.1	22.6	15.3
	**105**	11.8	16.9	11.7	2.9	2.8	7.0	5.0	6.7	5.6	7.0	5.6	8.0	7.6
	**111**	12.9		3.3										8.1
	**114**	20.3	22.2	20.9	6.8	2.4	5.8	8.6	18.4	9.1	13.3	20.3	17.8	13.8
	**121**	11.5	15.7	10.1	6.5	4.0	4.5	8.3	19.4		13.1	16.7	14.8	11.3
	**125**	21.6	26.6	30.9										26.4
**Heated**	**102**	4.2	3.7	5.6										4.5
	**104**	28.4	24.5	19.0	7.1	6.1	15.2	12.5	23.9	16.2	14.4	14.0	19.1	16.7
	**112**	11.8	18.7	15.9	12.0	10.2	12.5	7.3	22.4	18.9	16.2	21.8	16.6	15.3
	**115**	5.1	12.7	5.1										7.6
	**122**	14.5	21.5	17.9	7.6	7.9	21.4	21.8	34.5		25.5	25.5		19.8
	**124**	4.2	8.1											6.2
**Means**														
**Control**		15.4	21.6	16.6	7.4	4.3	8.0	8.5	15.1	7.9	11.3	13.9	15.8	12.7
**Heated**		11.4	14.9	12.7	8.9	8.1	16.4	13.9	26.9	17.6	18.7	20.4	17.9	15.0
**Overall**		13.4	17.9	14.8	8.0	5.9	11.6	10.8	20.2	11.8	14.4	16.7	16.5	13.7

Table S6. Growth cessation (DOY; 80% of yearly growth completed) for control and soil-heated balsam fir trees over a 12-yr period (2010–2021).

**Trees**							**Years**							**Means**
		**2010**	**2011**	**2012**	**2013**	**2014**	**2015**	**2016**	**2017**	**2018**	**2019**	**2020**	**2021**	
**Control**	**101**	197	203	198	205	208	205	204	208	205	216	213	190	204
	**105**	197	199	189	192	211	204	205	218	207	220	194	179	201
	**111**	191		200										196
	**114**	199	202	187	191	209	200	204	202	205	215	196	195	200
	**121**	198	202	198	192	227	202	204	215		207	205	195	204
	**125**	199	207	187										198
**Heated**	**102**	190	234	199										208
	**104**	190	194	199	192	208	202	204	201	204	209	193	181	198
	**112**	191	194	197	191	206	199	200	197	195	200	193	187	196
	**115**	191	199	188										193
	**122**	197	202	199	205	208	209	224	216		219	208		209
	**124**	198	202											200
**Means**														
**Control**		197	203	193	195	214	203	204	211	206	215	202	190	202
**Heated**		193	204	196	196	207	203	209	205	200	209	198	184	200
**Overall**		195	204	195	195	211	203	206	208	203	212	200	188	201

Table S7. Growth cessation (DOY; 90% of yearly growth completed) for control and soil-heated balsam fir trees over a 12-yr period (2010–2021).

**Trees**		**Years**												**Means**
		**2010**	**2011**	**2012**	**2013**	**2014**	**2015**	**2016**	**2017**	**2018**	**2019**	**2020**	**2021**	
**Control**	**101**	216	217	219	215	245	224	209	217	217	231	218	206	220
	**105**	207	209	224	247	267	215	264	236	210	248	248	182	230
	**111**	194		227										211
	**114**	217	220	199	192	245	203	228	217	246	231	216	216	219
	**121**	251	220	224	216	254	225	239	225		216	213	206	226
	**125**	215	219	198										211
**Heated**	**102**	269	241	223										244
	**104**	194	203	224	221	228	204	252	217	207	232	218	195	216
	**112**	199	203	219	216	208	204	227	208	205	216	208	195	209
	**115**	252	203	225										227
	**122**	207	231	219	216	244	222	235	230		230	216		225
	**124**	268	233											251
**Means**														
**Control**		217	217	215	218	253	217	235	224	224	232	224	203	222
**Heated**		232	219	222	218	227	210	238	218	206	226	214	195	221
**Overall**		224	218	218	218	242	214	236	221	217	229	220	200	222

Table S8. Growing season duration (days; from 5% to 80% of yearly growth completed) for control and soil-heated balsam fir trees over a 12-yr period (2010–2021).

**Trees**		**Years**												**Means**
		**2010**	**2011**	**2012**	**2013**	**2014**	**2015**	**2016**	**2017**	**2018**	**2019**	**2020**	**2021**	
**Control**	**101**	52	49	48	61	52	57	50	48	52	51	50	45	51
	**105**	51	42	42	44	73	53	48	55	42	52	31	33	47
	**111**	35		45										40
	**114**	54	48	44	58	45	53	54	52	53	53	46	49	51
	**121**	53	48	55	59	90	52	46	64		45	54	49	56
	**125**	54	53	44										50
**Heated**	**102**	45	65	48										53
	**104**	45	36	53	39	45	52	49	46	48	47	30	36	44
	**112**	46	40	51	48	52	51	48	44	43	38	41	42	45
	**115**	46	41	42										43
	**122**	52	43	49	62	54	62	73	66		57	57		58
	**124**	53	43											48
**Means**														
**Control**		50	48	46	56	65	54	50	55	49	50	45	44	51
**Heated**		48	45	49	50	50	55	57	52	46	47	43	39	48
**Overall**		49	46	47	53	59	54	53	54	48	49	44	42	50

Table S9. Growing season duration (days; from 5% to 90% of yearly growth completed) for control and soil-heated balsam fir trees over a 12-yr period (2010–2021).

**Trees**							**Years**							**Means**
		**2010**	**2011**	**2012**	**2013**	**2014**	**2015**	**2016**	**2017**	**2018**	**2019**	**2020**	**2021**	
**Control**	**101**	71	63	69	71	89	76	55	57	64	66	55	61	66
	**105**	61	52	77	99	129	64	107	73	45	80	85	36	76
	**111**	38		72										55
	**114**	72	66	56	59	81	56	78	67	94	69	66	70	70
	**121**	106	66	81	83	117	75	81	74		54	62	60	78
	**125**	70	65	55										63
**Heated**	**102**	124	72	72										89
	**104**	49	45	78	68	65	54	97	62	51	70	55	50	62
	**112**	54	49	73	73	54	56	75	55	53	54	56	50	59
	**115**	107	45	79										77
	**122**	62	72	69	73	90	75	84	80		68	65		74
	**124**	123	74											99
**Means**														
**Control**		70	62	68	78	104	68	80	68	68	67	67	57	71
**Heated**		87	60	74	71	70	62	85	66	52	64	59	50	69
**Overall**		78	61	71	75	89	65	82	67	61	66	63	55	70

Table S10: Mixed between subjects (control/heated) and within subjects (years) analyze of variance of the trimmed means of parameters used to characterize growth phenology for 12 balsam fir trees over the 2010–2021 period, including the 5 trees that not survived from the combined drought and defoliation event occurring in 2012.

Effect	Onset	Cessation			Duration			Total	Rate
	5%	80%	90%	95%	80%	90%	95%		
Treatment	0.3	1.8	1.2	6.2*	2.7	1.5	7.5*	2.4	5.6*
Year	120***	38.0***	15.5***	129***	3.3*	3.3*	22.8***	3.1*	9.9***
Interaction	5.7**	0.7	1.8	102***	1.4	0.5	3.8*	1.0	1.2

Significance: * < 0.1, ** < 0.01, *** < 0.001

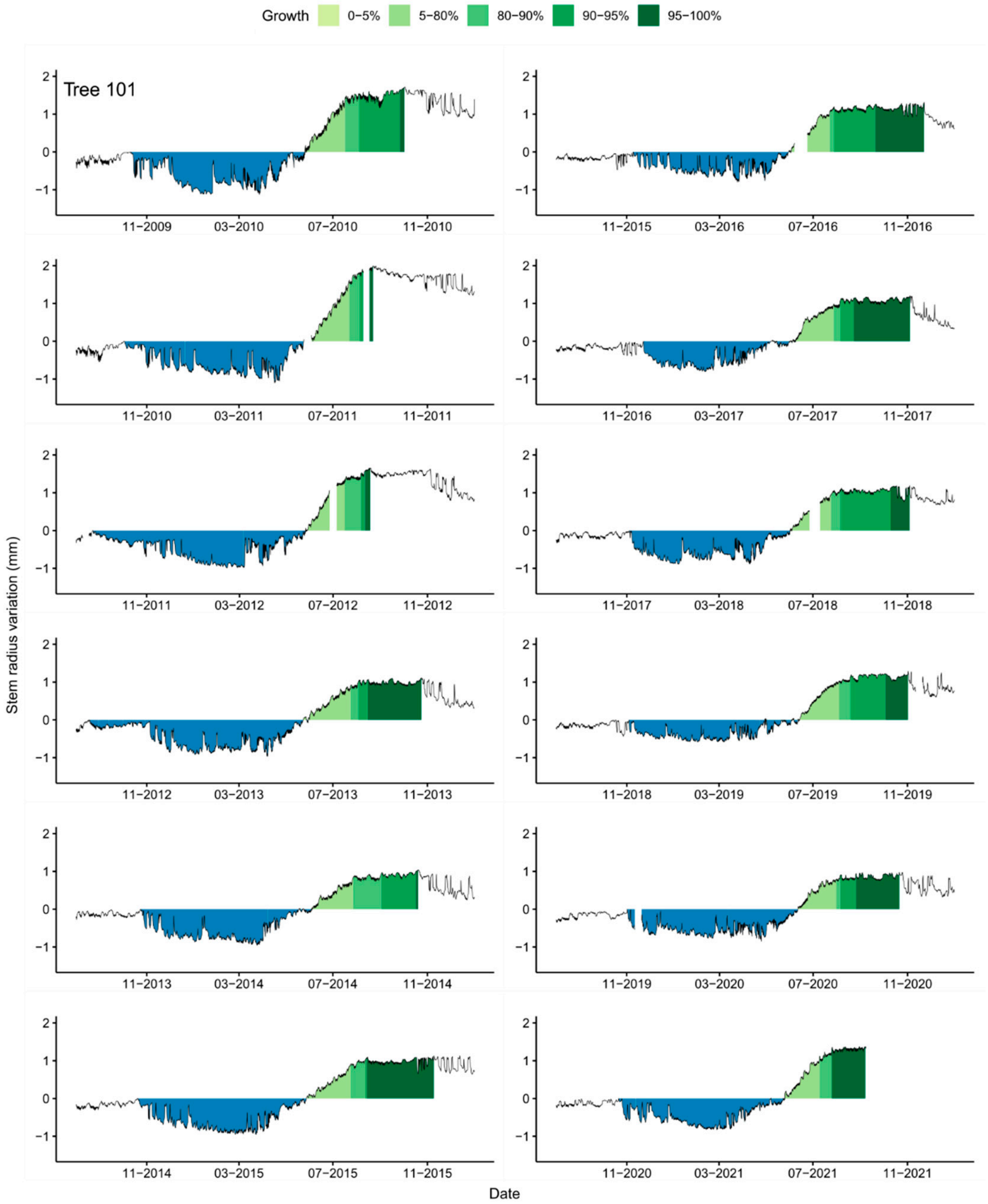

Figure S1: Characterization of growing season from stem radius variations of a balsam fir tree (no. 101) recorded by a dendrometer at a temporal resolution of 15 min over a twelve-year period. The green colors correspond to the DOY when 5%, 80%, 90%, 95% and 100% of the total annual growth is completed, while the blue area corresponds to the shrinking of the tree diameter in winter due to dehydration.

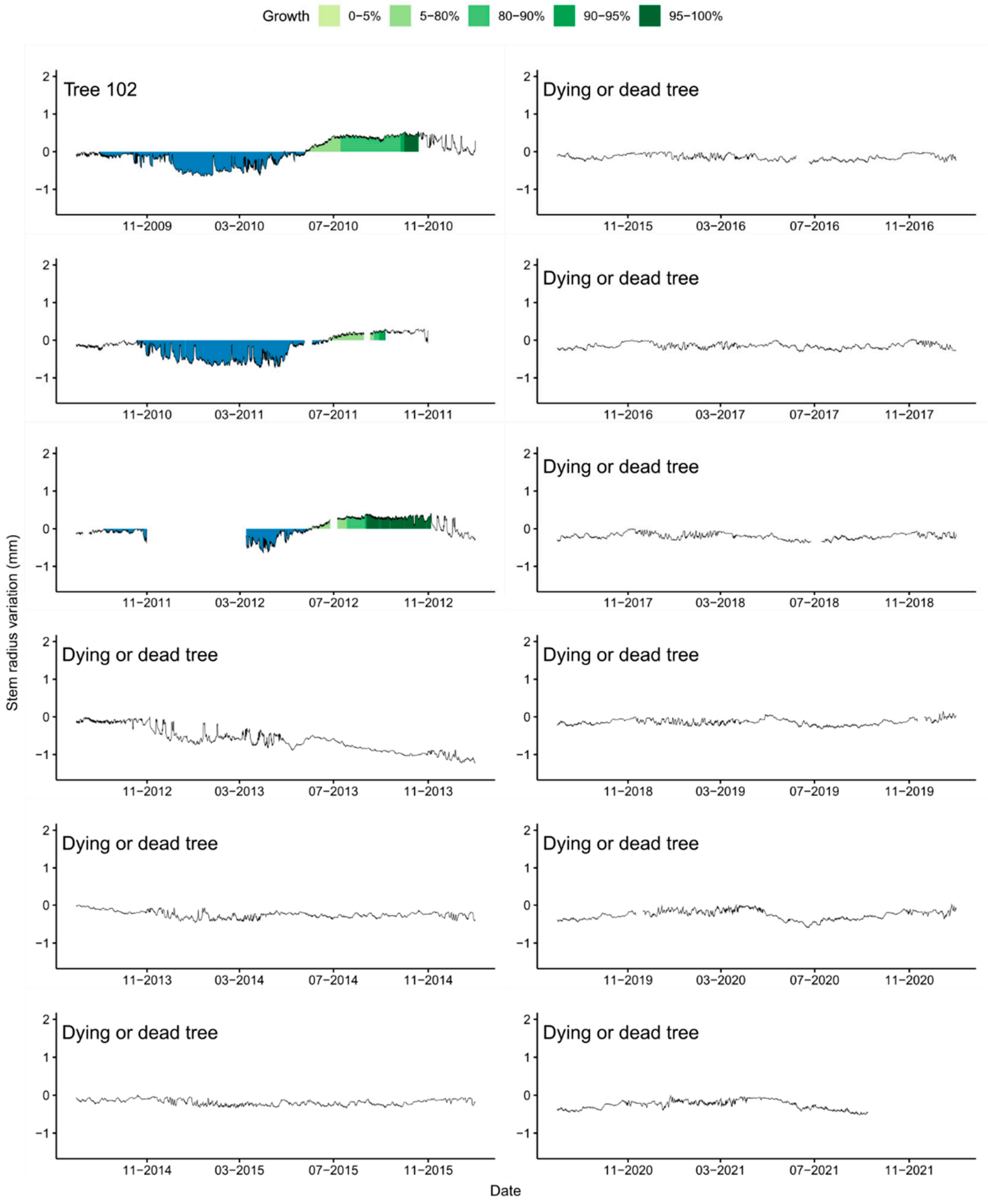

Figure S2: Characterization of growing season from stem radius variations of a balsam fir tree (no. 102) recorded by a dendrometer at a temporal resolution of 15 min over a twelve-year period. The green colors correspond to the DOY when 5%, 80%, 90%, 95% and 100% of the total annual growth is completed, while the blue area corresponds to the shrinking of the tree diameter in winter due to dehydration.

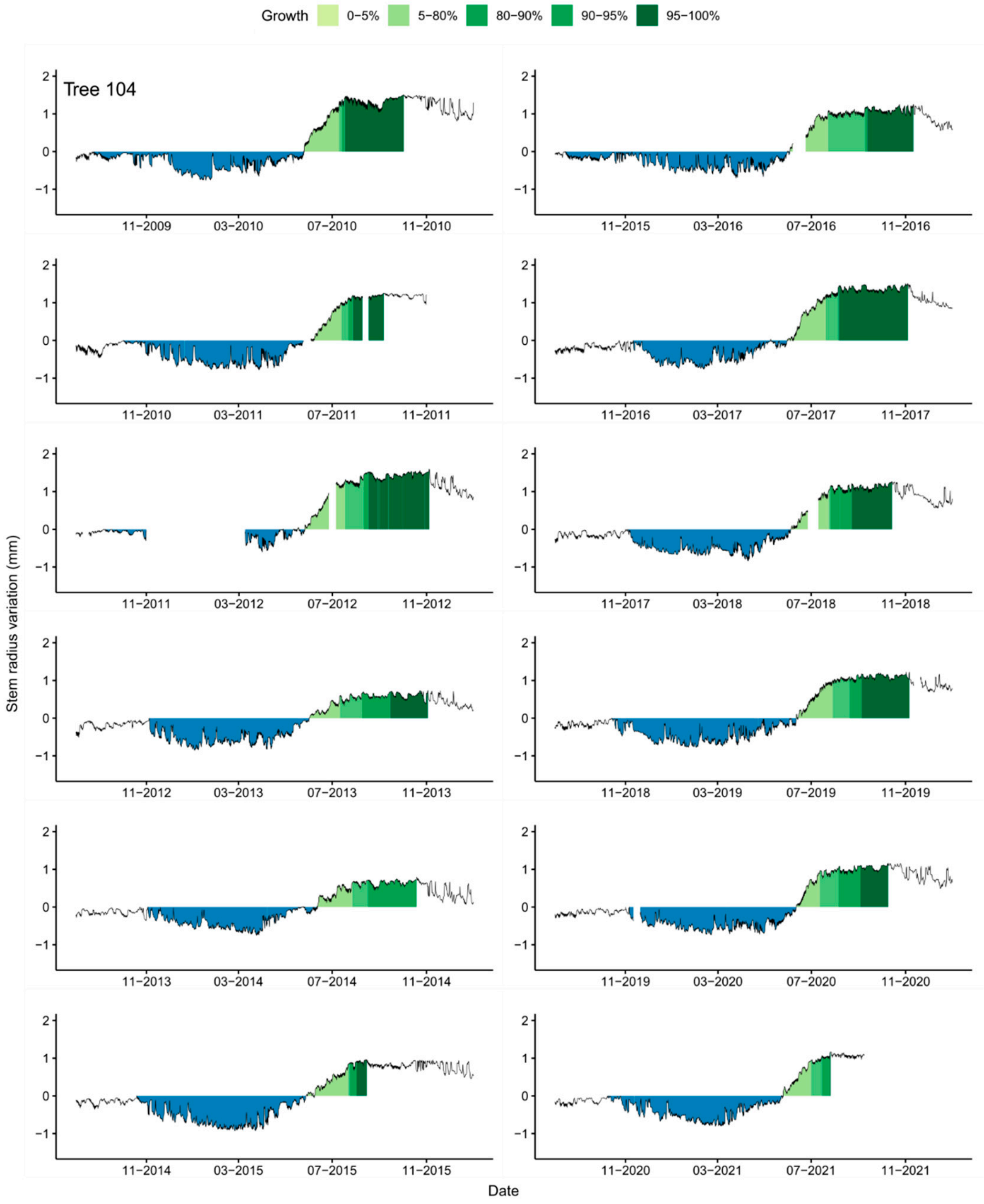

Figure S3: Characterization of growing season from stem radius variations of a balsam fir tree (no. 104) recorded by a dendrometer at a temporal resolution of 15 min over a twelve-year period. The green colors correspond to the DOY when 5%, 80%, 90%, 95% and 100% of the total annual growth is completed, while the blue area corresponds to the shrinking of the tree diameter in winter due to dehydration.

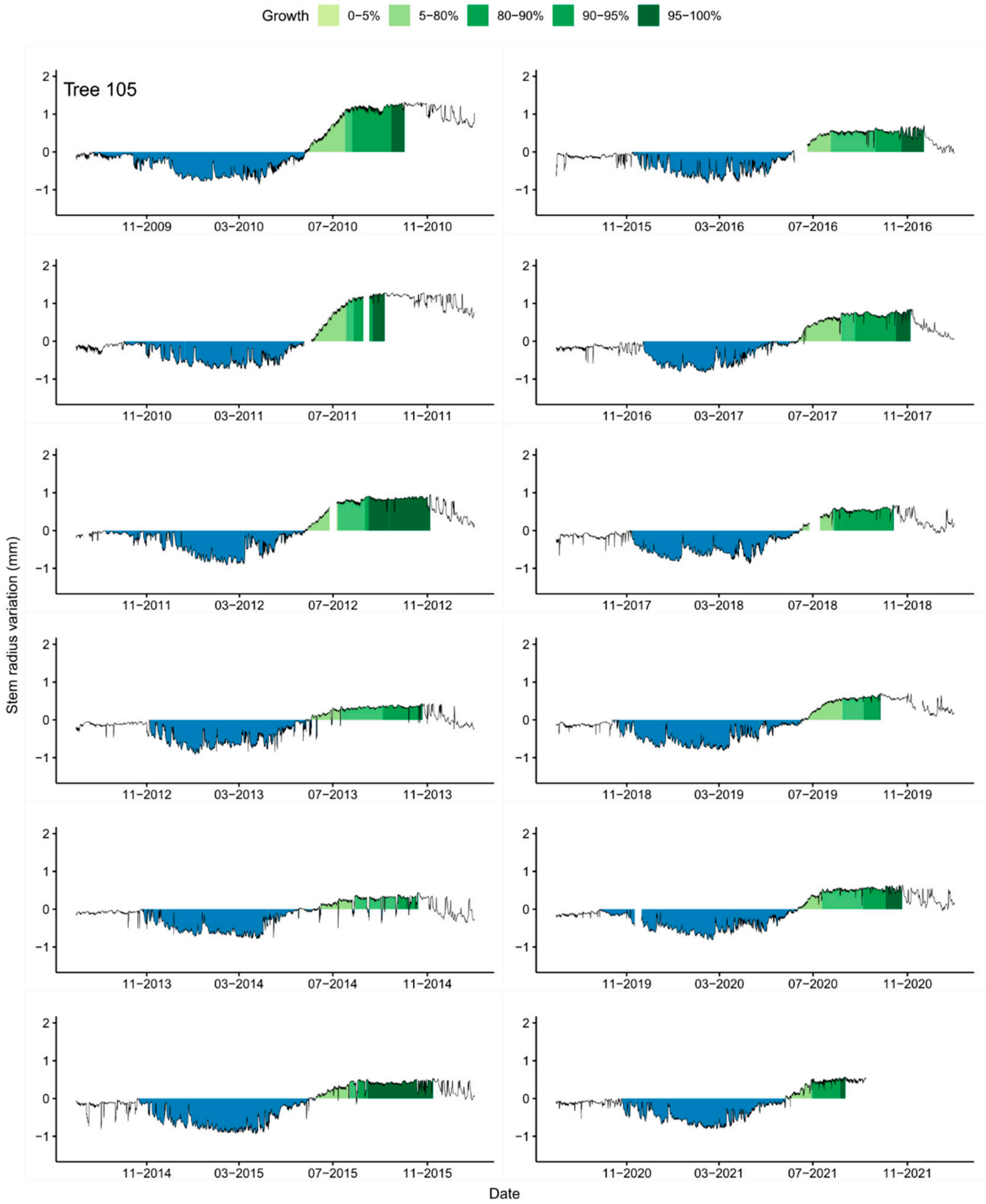

Figure S4: Characterization of growing season from stem radius variations of a balsam fir tree (no. 105) recorded by a dendrometer at a temporal resolution of 15 min over a twelve-year period. The green colors correspond to the DOY when 5%, 80%, 90%, 95% and 100% of the total annual growth is completed, while the blue area corresponds to the shrinking of the tree diameter in winter due to dehydration.

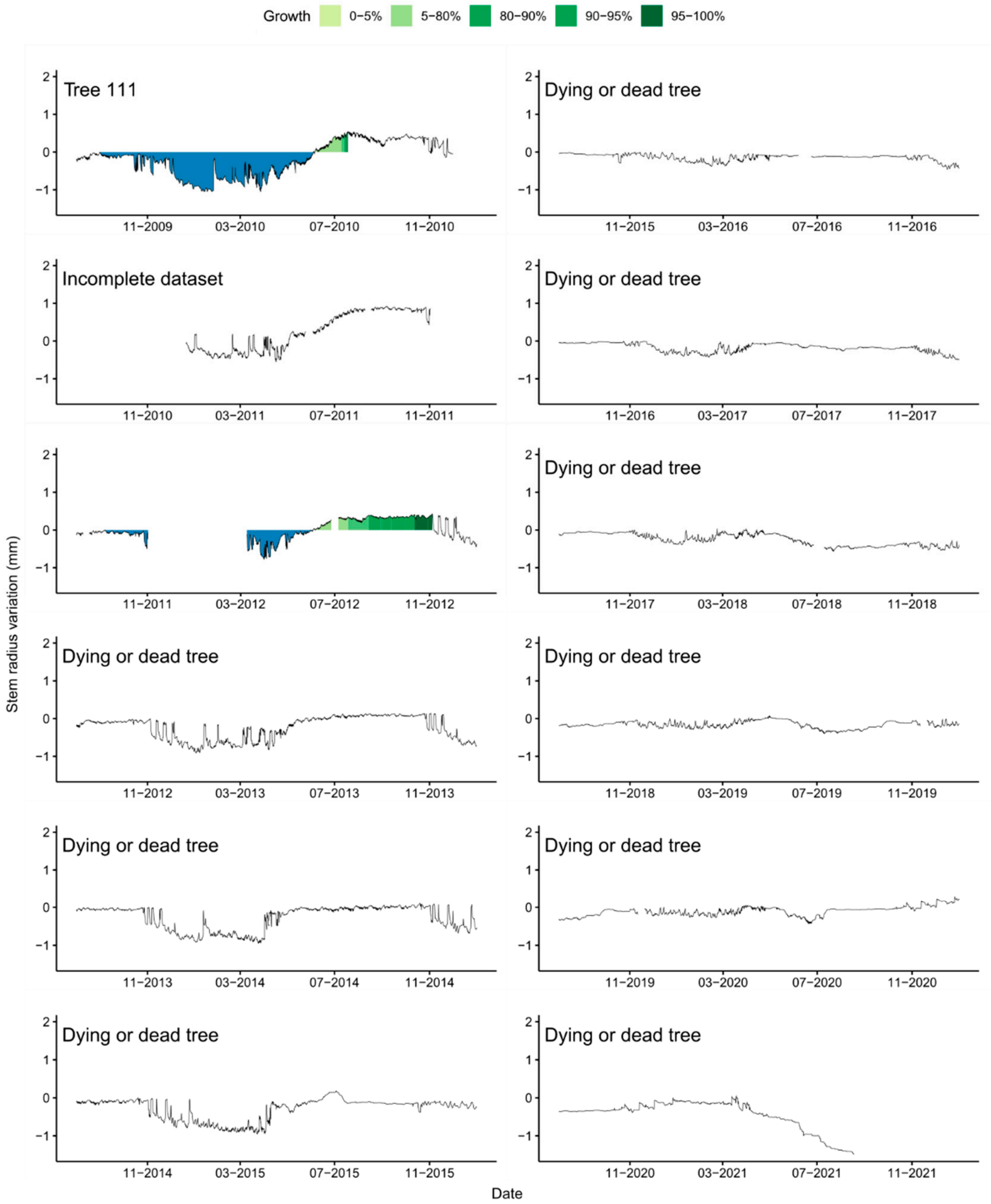

Figure S5: Characterization of growing season from stem radius variations of a balsam fir tree (no. 111) recorded by a dendrometer at a temporal resolution of 15 min over a twelve-year period. The green colors correspond to the DOY when 5%, 80%, 90%, 95% and 100% of the total annual growth is completed, while the blue area corresponds to the shrinking of the tree diameter in winter due to dehydration.

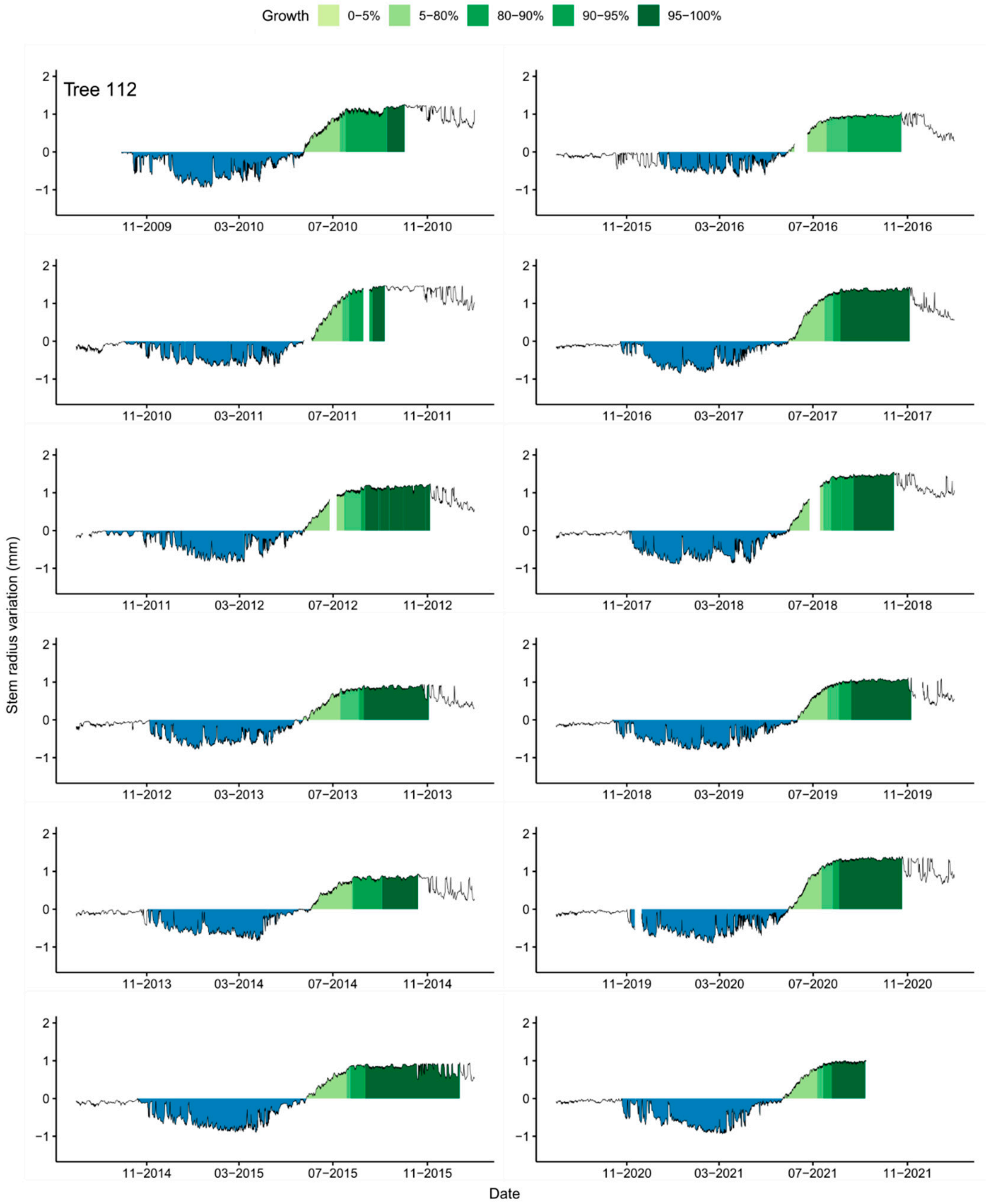

Figure S6: Characterization of growing season from stem radius variations of a balsam fir tree (no. 112) recorded by a dendrometer at a temporal resolution of 15 min over a twelve-year period. The green colors correspond to the DOY when 5%, 80%, 90%, 95% and 100% of the total annual growth is completed, while the blue area corresponds to the shrinking of the tree diameter in winter due to dehydration.

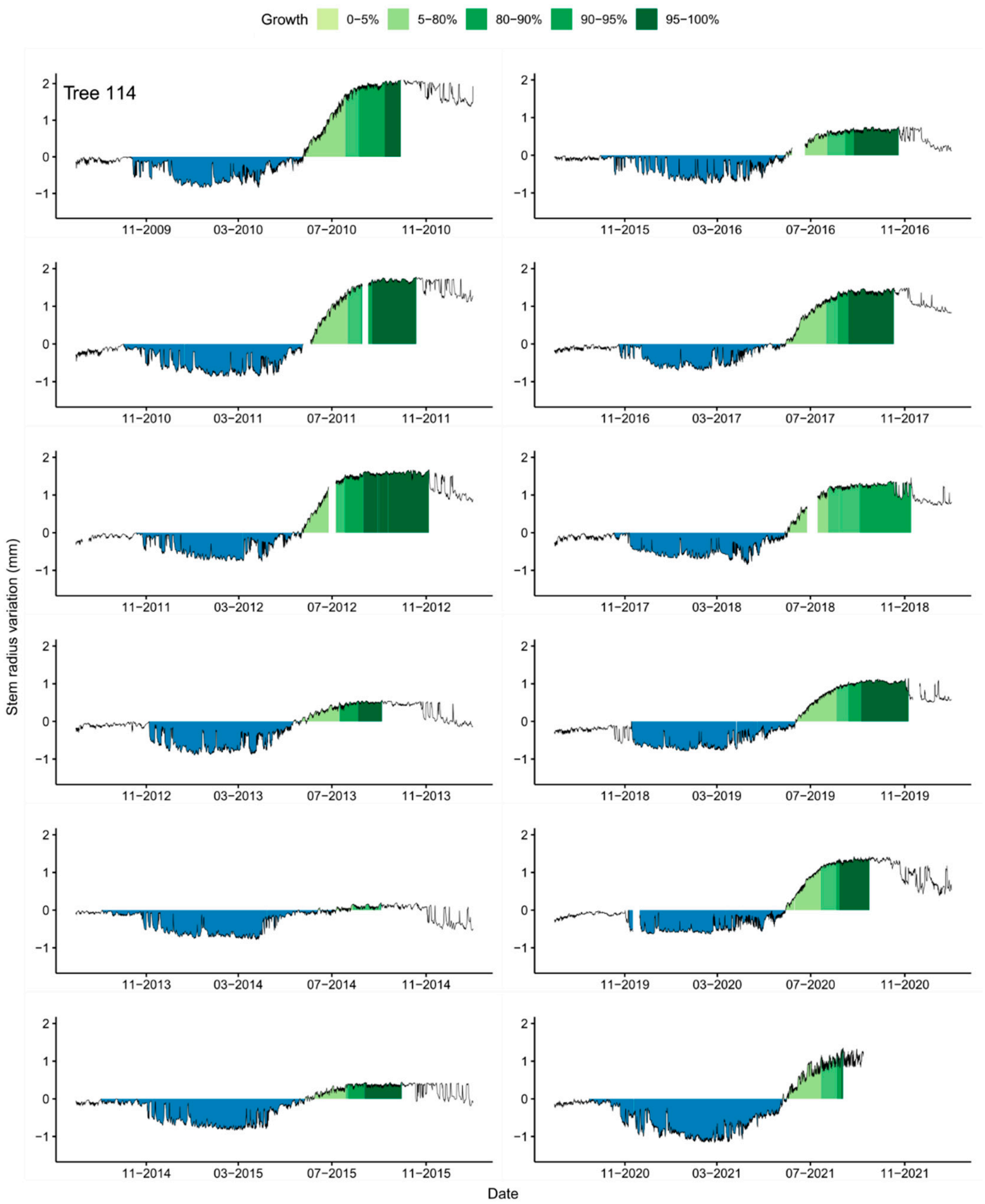

Figure S7: Characterization of growing season from stem radius variations of a balsam fir tree (no. 114) recorded by a dendrometer at a temporal resolution of 15 min over a twelve-year period. The green colors correspond to the DOY when 5%, 80%, 90%, 95% and 100% of the total annual growth is completed, while the blue area corresponds to the shrinking of the tree diameter in winter due to dehydration.

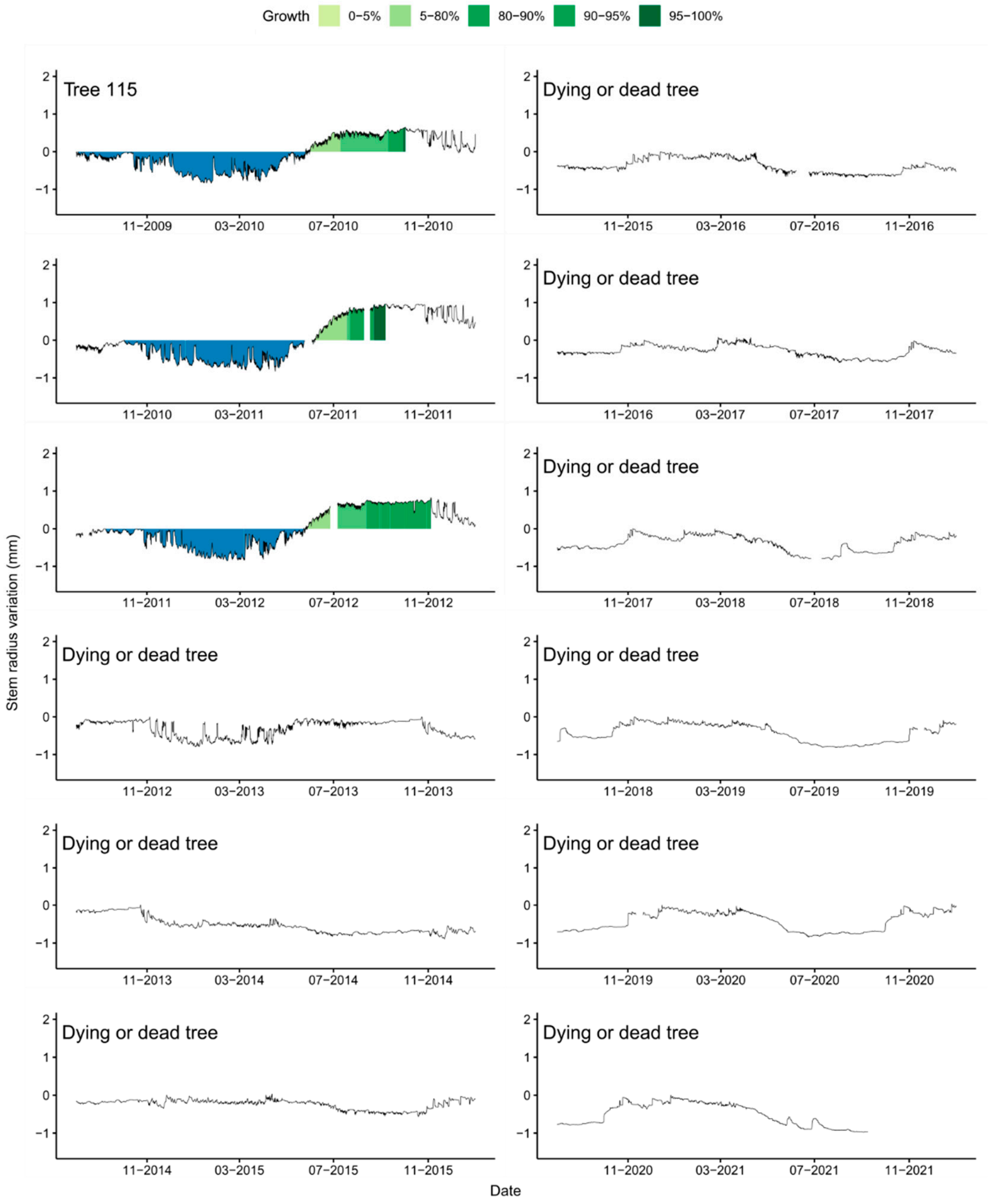

Figure S8: Characterization of growing season from stem radius variations of a balsam fir tree (no. 115) recorded by a dendrometer at a temporal resolution of 15 min over a twelve-year period. The green colors correspond to the DOY when 5%, 80%, 90%, 95% and 100% of the total annual growth is completed, while the blue area corresponds to the shrinking of the tree diameter in winter due to dehydration.

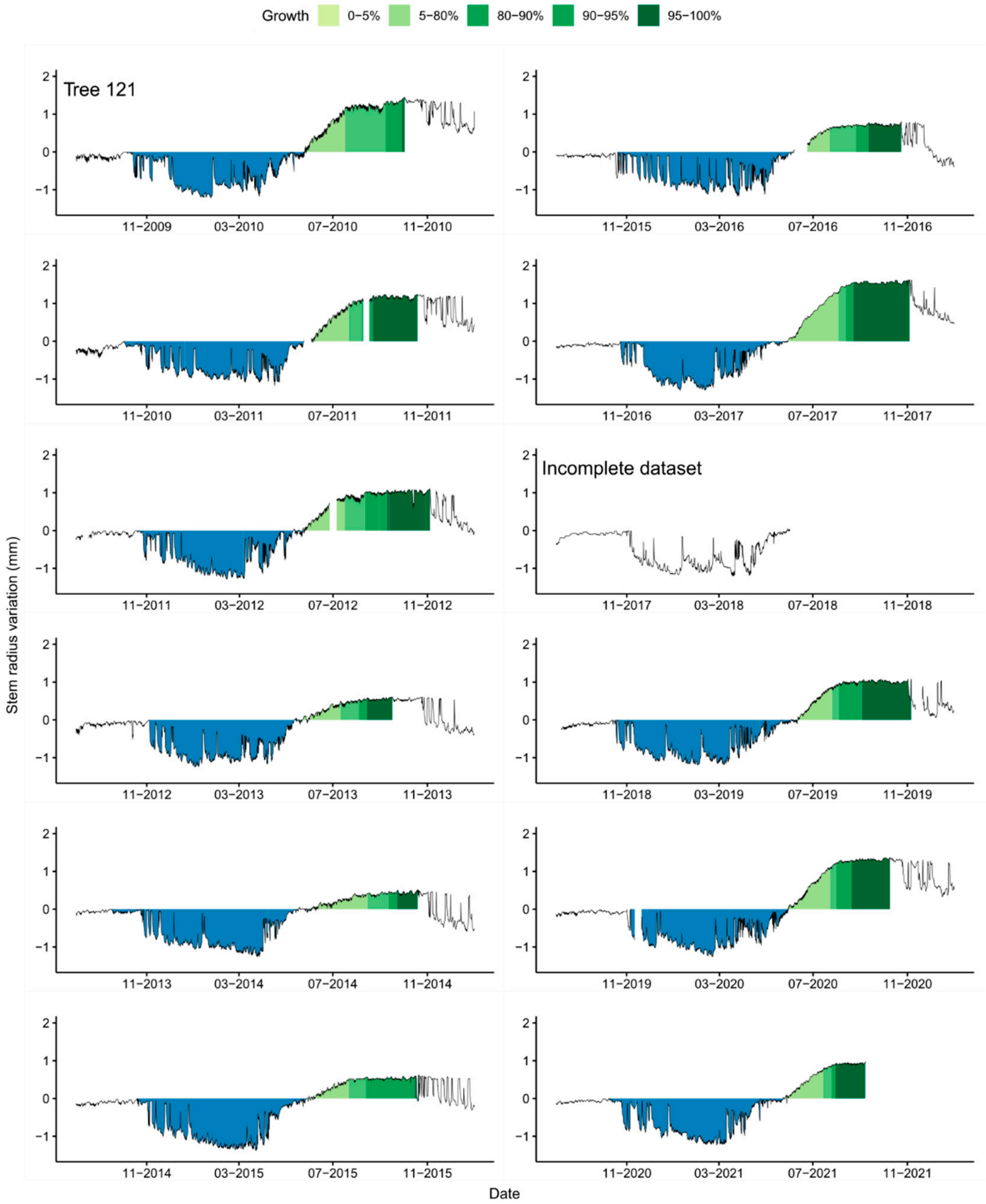

Figure S9: Characterization of growing season from stem radius variations of a balsam fir tree (no. 121) recorded by a dendrometer at a temporal resolution of 15 min over a twelve-year period. The green colors correspond to the DOY when 5%, 80%, 90%, 95% and 100% of the total annual growth is completed, while the blue area corresponds to the shrinking of the tree diameter in winter due to dehydration.

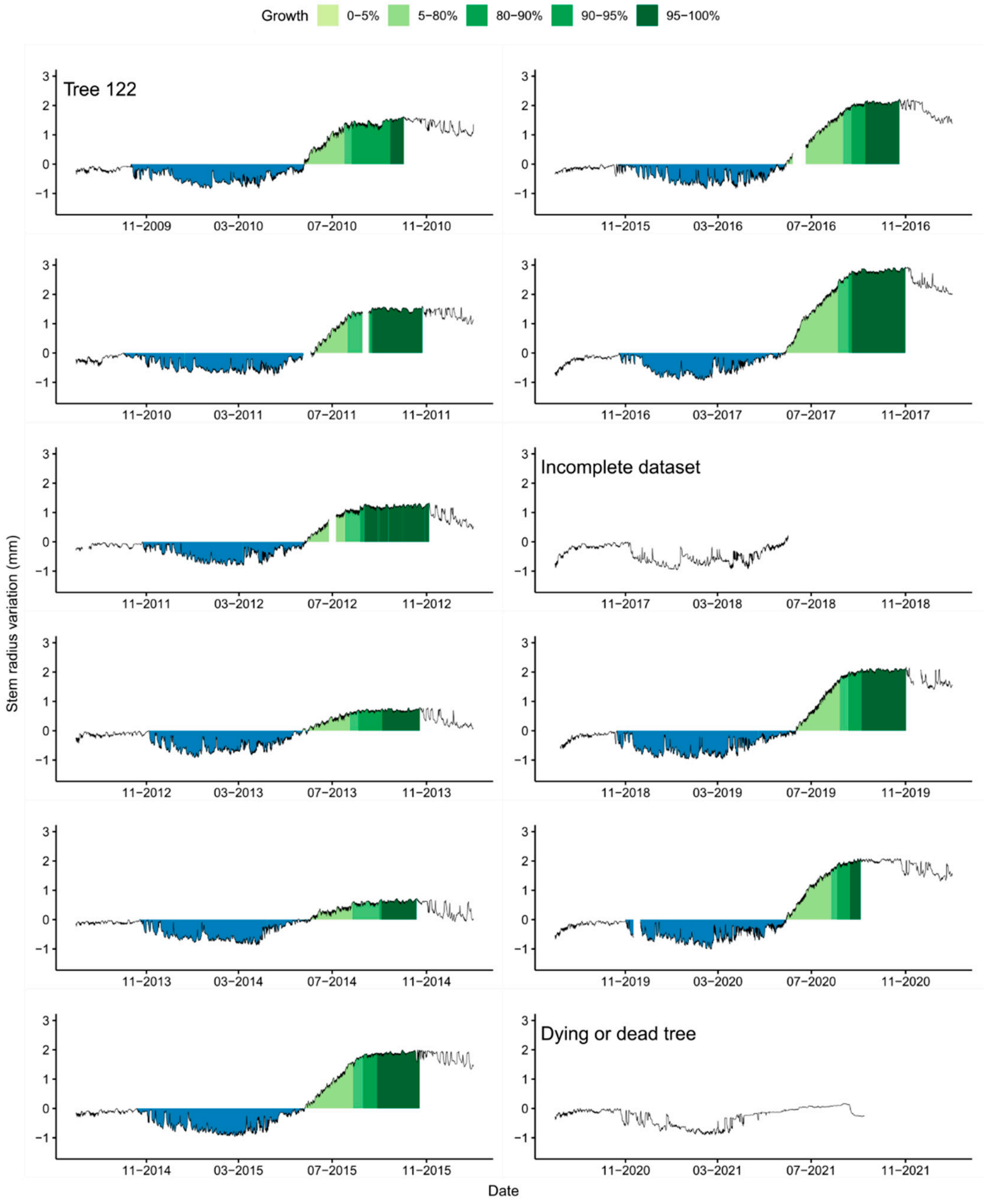

Figure S10: Characterization of growing season from stem radius variations of a balsam fir tree (no. 122) recorded by a dendrometer at a temporal resolution of 15 min over a twelve-year period. The green colors correspond to the DOY when 5%, 80%, 90%, 95% and 100% of the total annual growth is completed, while the blue area corresponds to the shrinking of the tree diameter in winter due to dehydration.

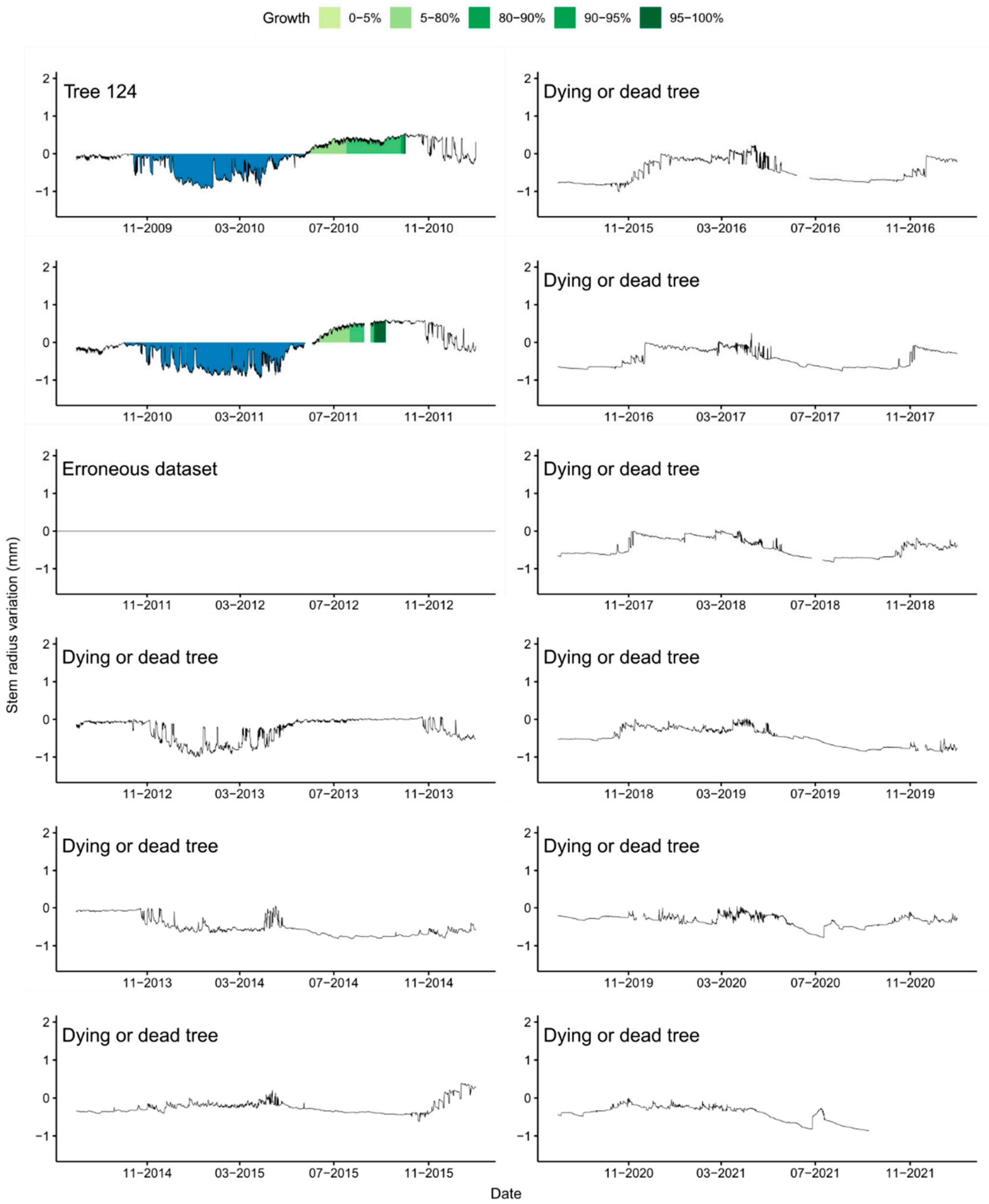

Figure S11: Characterization of growing season from stem radius variations of a balsam fir tree (no. 124) recorded by a dendrometer at a temporal resolution of 15 min over a twelve-year period. The green colors correspond to the DOY when 5%, 80%, 90%, 95% and 100% of the total annual growth is completed, while the blue area corresponds to the shrinking of the tree diameter in winter due to dehydration.

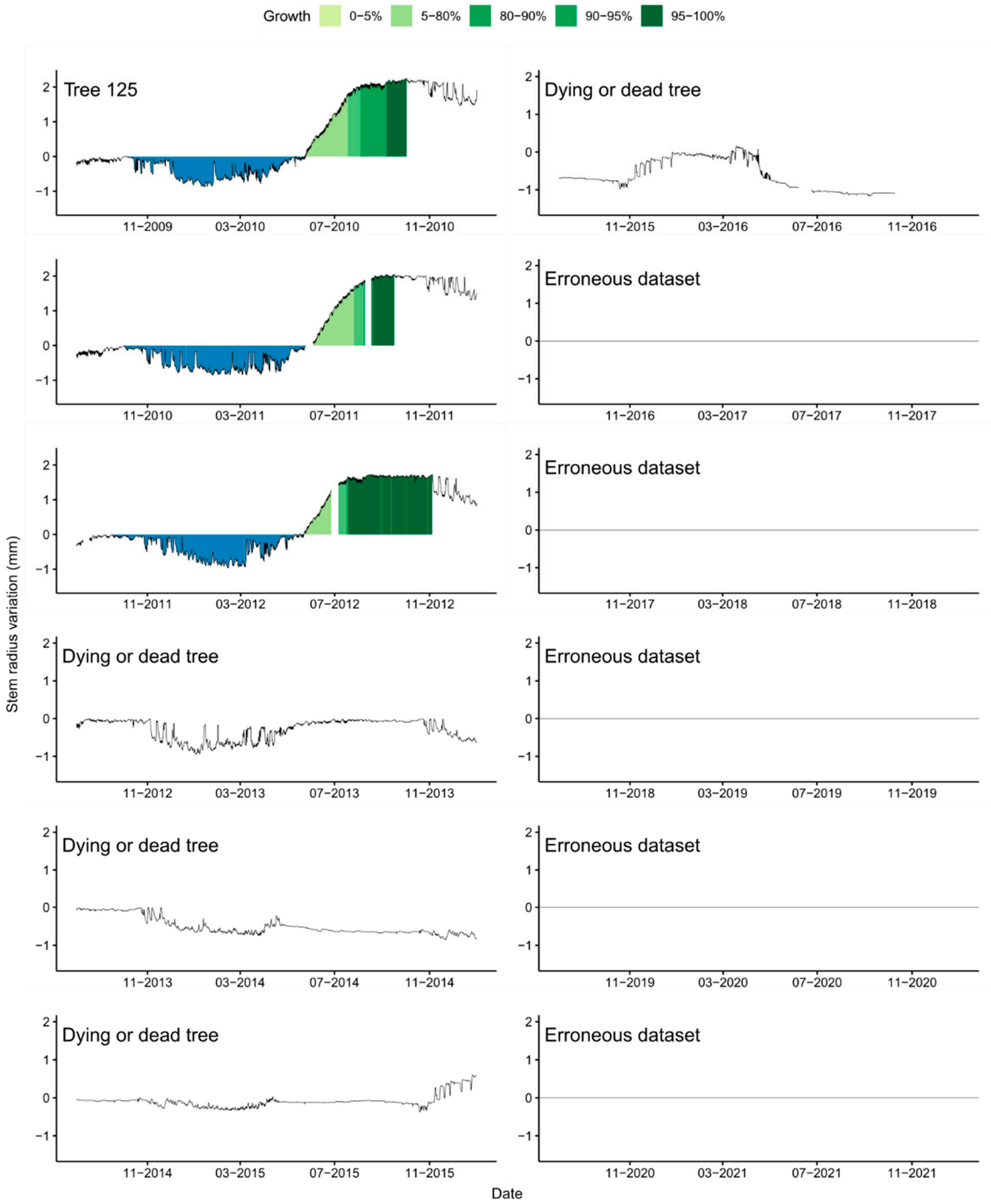

Figure S12: Characterization of growing season from stem radius variations of a balsam fir tree (no. 125) recorded by a dendrometer at a temporal resolution of 15 min over a twelve-year period. The green colors correspond to the DOY when 5%, 80%, 90%, 95% and 100% of the total annual growth is completed, while the blue area corresponds to the shrinking of the tree diameter in winter due to dehydration.
